# Perceptions of patients and their relatives about schadenfreude towards doctors

**DOI:** 10.1016/j.heliyon.2024.e32983

**Published:** 2024-06-20

**Authors:** Fatih Yıldırım, Zeynep Çakır, Sefa Özdemir, İnci Yılmazlı Trout, Atıf Bayramoğlu, Oğuzhan Ekinci, Serap Atasever Belli, İkram Yusuf Yarbaşı, Muhammet Mutlu, Rıdvan Akın, Burcu Yaşar, Seda Kayapalı Yıldırım, Ezgi Kaşdarma, Begüm Yılmazcan

**Affiliations:** a Dept. of Business Administration, Faculty of Economics and Administrative Sciences, Erzurum Technical University, Erzurum, Turkey; b Dept. of Emergency Medicine, Faculty of Medicine, Erzurum Technical University, Erzurum, Turkey; c Dreeben School of Education, University of the Incarnate Word, San Antonio, Texas, USA; d Internal Medicine, Faculty of Medicine, Alanya Alaaddin Keykubat University, Alanya, Turkey; e Dept. of Psychology, Faculty of Letters, Erzurum Technical University, Erzurum, Turkey; f Dept. of English Language and Literature, Faculty of Letters, Erzurum Technical University, Erzurum, Turkey; g Dept. of Econometrics, Faculty of Economics and Administrative Sciences, Erzurum Technical University, Erzurum, Turkey; h Dept. of Commercial Law, Faculty of Economics and Administrative Sciences, Erzurum Technical University, Erzurum, Turkey; i Dept. of Labor Economics and Industrial Relations, Faculty of Economics and Administrative Sciences, Atatürk University, Erzurum, Turkey; j Dept. of Psychology, Social Psychology, Faculty of Science and Letters, Kütahya Dumlupınar University, Kütahya, Turkey; k Independent Scholar, Turkey

**Keywords:** Violence, Violence in healthcare, Violence against doctors, Schadenfreude

## Abstract

**Background:**

Violence in healthcare is a global issue that healthcare professionals experience. The concerning increase in violent incidents in Turkiye particularly makes it a significant problem to explore by examining the underlying psychological factors. In this sense, this study focuses on the concept of Schadenfreude, the malicious joy of someone else's misfortune, towards doctors, which is an under-researched topic in healthcare violence. Particularly, there is a lack of research on patients' and relatives' perceptions of doctors.

**Objective:**

This study aims to determine the level of schadenfreude in Turkish society towards the violence experienced by doctors and to develop a model revealing the underlying causes.

**Methods:**

Using a convenience sampling method, we recruited 402 participants, who are not first-degree relatives of healthcare professionals, for this quantitative study. For data collection, we developed a survey instrument to measure the level of schadenfreude and six different psychological factors including empathy, sympathy, anger, aggression, and deservingness. For data analysis, we used structural equation modeling.

**Results:**

The results showed that the lower the levels of empathy and sympathy towards doctors were, the higher the levels of both schadenfreude and aggression were. Envy had no significant effect on either schadenfreude or aggression, while deservingness directly affected aggression. The perceptions of participants regarding doctors that they deserve violence increased their aggression levels. Schadenfreude had a positive and significant effect on anger and aggression.

**Implications:**

The examination of underlying factors of violence towards doctors points to a lack of mutual understanding between patients and doctors. The results of this study indicate a need for increasing empathy towards health professionals by creating societal awareness of their experiences. Local authorities and healthcare organizations can create environments that bring together the public and health professionals to share their experiences with each other or conduct campaigns to draw public attention to the issue. Moreover, training sessions on effective communication can be offered for health professionals to help improve patient-doctor relationships and healthcare outcomes.

## Highlights

• This study explores Schadenfreude for the first time in healthcare to inform policy and practice regarding violence in healthcare.
• This study uniquely addresses all five dimensions of schadenfreude together to explore violence against doctors in Turkiye, unlike past research.
• Violence has led many doctors to leave Turkey, resulting in a loss of qualified labor. To develop a new perspective, our research examined healthcare violence and various related concepts.
• Exploration of factors related to the concept of schadenfreude through structural equation modeling after having tested the explanatory and confirmatory factor analyses.

## 1 Introduction

### 1.1 Violence in healthcare

Healthcare violence is a prevalent global issue with employees in the healthcare sector facing a 16-fold higher risk of being exposed to violence than those in other sectors [1,2] and 50 % of healthcare professionals experiencing verbal violence globally and 25 % being subjected to physical violence at least once in their careers [[2], [3], [4]]. The World Health Organization (WHO) defines violence as the intentional use or threat of physical force against oneself, another person, or a group or community that results in or is likely to result in injury, death, psychological harm, developmental delay, or deprivation [5]. Violence in healthcare is defined as any incident involving verbal abuse, threatening behavior, economic abuse, or physical or sexual assault by patients and their relatives that puts a healthcare professional at risk [6,7].

Recent studies show a growing trend of violence in healthcare in Turkiye [8], and doctors constitute the group of health professionals who experience violent incidents the most (48.65 %) [2]. According to the Grand National Assembly of Turkiye (TBMM) Commission Report, patients' relatives are responsible for 91 % of healthcare-related violence, with emergency rooms accounting for 79 % of these cases. According to aforementioned report, the causes of violence in the health sector include the interaction between the parties, organizational factors, environmental factors, and social variables [9]. Other reasons include healthcare institutions, healthcare workers, and patients and patient relatives as well as exposure to news and publications against healthcare professionals, staff shortages at hospitals, and the belief in unfair treatment among patients and their relatives [8]. Moreover, several psychological factors such as deservingness, empathy, and envy have been revealed to have effects on violence [[10], [11], [12]]. These underlying psychological factors lead us to the emotion of ‘schadenfreude,’ which refers to finding joy in someone else's misfortune and is a concept not previously explored in healthcare violence. According to Frijda, individuals are more inclined to take action when accompanied by a shared emotion and idea [13]. In other words, individuals tend to exhibit a behavioral tendency to punish others due to the feeling of resentment [14].

### 1.2 The concept of schadenfreude

Psychological factors that may affect passive bystanders as well as perpetrators and victims include the feeling of satisfaction from harming another. Although scientific claims about this emotion date back to the ancient Greeks, researchers continue to use the German term schadenfreude because no English term fully captures its meaning. Schadenfreude, which means malicious joy at the misfortune of another, is a combination of the German words schaden, meaning harm, and freude, meaning joy [15]. Various studies have been undertaken on the reasons why people feel this way. For example, a harmful event may be welcomed by some observers as punishment for a normative violation. The misfortune of someone perceived to have an unfair advantage can lead to complacency in some observers. The status and advantages of the person experiencing misfortune create a feeling of jealousy in others, and the sadness of these people can create a feeling of pleasure in them [16].

Many studies that associate the emergence of Schadenfreude with the social comparison between the parties have focused on the role of one party's previous disadvantage [17]. According to Steinbeis and Singer, however, any advantageous situation can spontaneously elicit Schadenfreude [18]. Although the concept of Schadenfreude has not been examined much in this alternative, it is very important to better understand the concept [19].

In this respect, earlier studies have shown that people feel schadenfreude in various conditions, such as when they think that the person who has experienced a bad event deserves it, when the person who has experienced misfortune is someone who is envied, or when the feeling of sympathy for the target person is low [[20], [21], [22]]. Further, a lack of empathy, high levels of aggression, and anger could also yield a feeling of schadenfreude [23,24]. To sum up, concepts such as deservingness, envy, sympathy, and empathy have been identified in the literature as the causes of schadenfreude. However, how or to what extent these concepts differ from one another in affecting the level of schadenfreude has not been explored yet. Thus, integrating these concepts under the frame of the schadenfreude model through the current study could provide unique and fruitful insights into the underlying causes of schadenfreude regarding violence in the healthcare field.

In this respect, with the concept of schadenfreude, which focuses on the passive spectator (society) as well as the perpetrator and the victim, this study aims to uncover society's perspective on this type of violence and to provide scientific support to policymakers. This study aims to offer unique insights into the perceptions of patients and their relatives towards doctors, which have not been explored yet. Further, while data on scientific measures of concepts such as anger and envy for individual physicians are not yet available in the literature, presenting them collectively through a model created with six different psychological concepts could set forth a thorough picture of the causes and effects of psychological variables on one another in one model and may be beneficial by providing important data for scientific research.

### 1.3 Deservingness

One of the factors that makes someone feel happy about a bad incident someone else has experienced is the observer's belief that the victim deserved the bad incident. Feather defined the emotions that occur as a response to events based on whether the bad events that happen to oneself or someone else are deserved or undeserved [25]. When a target experiences a negative incident that they do not deserve, it is likely that the observer will feel empathy for that person. It is also possible that when a target experiences a well-deserved negative incident, observers are likely to enjoy the situation [26]. The more a person feels responsible for the bad event that happened to them, the more that situation is perceived as deserved, and the more people feel schadenfreude [27]. Providing little to no information to patients and their relatives, not answering their questions, healthcare providers not being friendly or courteous, or poor performance of healthcare providers may create a perception in society that doctors deserve violence [8].

### 1.4 Envy

Envy is felt when people perceive various advantages in others by comparing themselves to them. It is seen that people not only envy the individual goals they set for themselves but also harbor an envious prejudice against them. It is sometimes possible for this stated envious prejudice to develop into a feeling of schadenfreude. Particularly, those with high status are more likely than others to experience envy, and others are more likely to react with happiness when these people suffer misfortune [21]. There are studies revealing that individuals feel stronger jealousy towards the high-status hero and are happy about his misfortune [[28], [29], [30]]. Specifically, the perception about doctors as heros as a result of saving lives as part of their job, their perception of the high status of their profession, and their income above the social average can lead to envy of doctors.

### 1.5 Sympathy

One of the factors that determines people's reactions to the negative event that happened to the victim is the level of sympathy they feel for the target person [27]. A lack of sympathy in the audience for the target person experiencing misfortune leads to a sense of pleasure in the sadness of others [22]. Therefore, it can be said that the level of sympathy and the emotion of schadenfreude have an inverse correlation. The concept of sympathy is defined as an emotion that involves an individual feeling sad or worried for the person who is exposed to a negative situation, pain, or misfortune experienced by someone else [31,32]. Studies have concluded that there is a significant and negative relationship between feeling happy and sympathy for an unfortunate event that happens to someone else [32,33]. In this context, it can be argued that an individual's level of sympathy is one factor that influences their perception of violence in health care.

### 1.6 Empathy

Empathy is defined as the process by which an individual puts themselves in the other person's shoes and views events, accurately perceives the other person's feelings and thoughts, and is able to communicate this situation to the other person. The concept of empathy can be examined in two subdimensions: emotional and cognitive empathy. The act of sharing another person's experience by feeling their emotions is known as emotional empathy [34]. In the emotional empathy process, the individual experiences the other person's emotions by focusing more on the emotions. However, cognitive empathy is the process of being able to recognize the other person's emotions without experiencing them. The process of an individual adopting the other person's perspective and the ability to see events through the other person's eyes is also related to cognitive empathy [34,35]. Empathy, an important part of social skills, is of great importance for positive social behavior and interpersonal communication. While empathetic individuals are less prone to violence, they tend to be helpful to others and have morally developed judgments [36]. Research shows that variables such as empathy influence schadenfreude [19]. Research on this topic also shows that as individuals' levels of empathy increase, their tendencies toward aggression and bullying decrease [[37], [38], [39]]. In this context, it can be argued that an individual's level of empathy is one of the factors influencing their perception of violence in health care.

### 1.7 Anger

Contemporary thinking on emotions suggests that emotions have two basic functions. These include the self-regulatory and communicative roles, which help individuals regulate their interpersonal relationships. Emotions such as anger, fear, and joy arise in response to an individual's evaluation of events in the external environment or cognitive events in the internal world. Therefore, they trigger behavioral responses. Emotions also help regulate social interactions. For example, the function of sadness is to signal to others that the individual needs help and relief. Anger, on the other hand, conveys expressions that indicate a conflict of personal interests and invite the other party to retreat [40]. Research on schadenfreude suggests that there is a strong relationship between the emotions of envy, anger, and schadenfreude. For example, individuals' internal feelings of inferiority led to anger toward superior and external groups [41]. Therefore, the feeling of revenge caused by anger leads to the expectation that negative events will happen to external groups that are perceived as superior. In other words, individuals who feel angry due to a sense of inferiority wish for the misfortune of the said superior or external group to alleviate their discomfort [24].

### 1.8 Aggression

Aggression is defined as any behavior designed to harm or injure another [42]. However, aggressive behavior must have a purpose, and the person exposed to aggressive behavior must make efforts to avoid or prevent this behavior [43]. As the most common type of aggression, physical aggression refers to the use of physical force in response to a feeling of being humiliated or insulted. Verbal aggression, another type of aggression, refers to verbal responses to a commanding attitude, criticism, humiliation, or violation of physical space [44]. Aggression is a concept that includes different dimensions, such as emotions, thoughts, and behaviors. However, this concept is also closely related to concepts such as anger, hostility, or violence [43]. Aggression may be an innate tendency or may be the result of personality traits, environmental conditions, and upbringing [44].

Reasons such as news against healthcare workers in the media, understaffed and overcrowded healthcare institutions, and inadequate equipment in healthcare institutions may be the cause of anger and aggression [8]. Moreover, considering the reasons related to the patient and the patient's relatives, reasons such as impatience by patients and their relatives, the patient's and/or relative's unwillingness to listen to explanations, the lack of education of the perpetrators of violence, and the patient's or relative's unwillingness to comply with hospital rules may contribute to anger and aggression. In addition, the polarizing effect of such news in the media on society can lead to the perception of healthcare professionals as “outsiders” and the development of a sense of schadenfreude toward them. Moreover, the previous research emphasizes the importance of the sense of deservingness and justice in their study examining the motivation for Schadenfreude [[45], [46], [47]]. The feeling of schadenfreude is said to lead an individual to act against and punish people they consider to be unfair due to the concerns about justice [47,48]. In this respect, schadenfreude has been suggested to motivate vengeful/hatred behavior [18,49].

The increasing rates of violence discussed in this section indicate that the psychological basis of the issue of violence in healthcare has not been examined widely. Uncovering the concept of schadenfreude by delving into all these aforementioned psychological factors is expected to facilitate social welfare and offer a crucial foundation for addressing the matter globally. Measuring social perceptions and taking steps to create the psychological infrastructure will allow for the formation of the basis for behavioral changes. Although the need for research that will transform resisting violence against doctors into a social value is clear, it is a difficult task to create cultural values, conduct multifaceted research, and transfer them to daily life. Therefore, determining the level of satisfaction with the violence to which health professionals are subjected and revealing the reasons for this, together with the dimensions of the concept of schadenfreude (deservingness, anger, envy, aggression, sympathy, empathy), stands out as the first way to create this cultural value.

### 1.9 Objectives and significance of the study

Although the concept of schadenfreude has been used in business or sociological analyses, it has not been widely addressed in the area of violence in healthcare. It is important to adapt the knowledge of this concept to the field of violence in healthcare and to provide policymakers with a scientific infrastructure in this context. In particular, concepts such as deservingness, envy, sympathy, and empathy have been identified in the international literature as causes of schadenfreude. With the concept of schadenfreude, which focuses on the passive spectator (society) as well as the perpetrator and the victim, this study aims to uncover society's perspective on this type of violence and to provide scientific support to policymakers. This study also aims to offer unique insights into the perceptions of patients and their relatives towards doctors, which have not been explored yet. Further, while data on scientific measures of concepts such as anger and envy for individual physicians are not yet available in the literature, presenting them collectively through a model of schadenfreude may yield a broader understanding of the connections between the aforementioned concepts and the concept of schadenfreude.

Considering the rise in the number of violent incidents in healthcare, it is of utmost importance to measure social perceptions through this research and to offer suggestions on how to take steps to create the psychological infrastructure that will form the basis for behavioral changes. It is essential to present an analysis of the current situation regarding the perception of schadenfreude towards healthcare professionals in society. Accordingly, there is a need for research that will establish the idea of not tolerating violence against doctors as a social value. However, it is a difficult task to create cultural values, conduct multifaceted research, and transfer them to daily life. Therefore, determining the level of satisfaction with the violence to which health professionals are subjected and revealing the reasons for this, together with the dimensions of the concept of schadenfreude (deservingness, anger, envy, aggression, sympathy, empathy, etc.), stands out as the first way to create this cultural value. By doing so, awareness will be raised to act against violence in health care. Thus, ways can be sought to increase the safety, motivation, attendance, commitment, and retention of healthcare workers, who are among the most effective human resources. In this respect, the current research brings forth unique insights into the body of knowledge in the healthcare field, as we used the novel concept of Schadenfreude for the first time in the healthcare field to inform policy and practice regarding addressing violence in healthcare. Further, it makes an original adaptation to healthcare by addressing the concept with its sub-dimensions (i.e., deservingness, anger, envy, aggression, sympathy, and empathy) together in research. Designing a model that has never been constructed for doctors has an innovative and original value in terms of adding a new dimension to the literature.

Our study serves as a pioneering mode for future studies on violence in other healthcare professions by focusing on doctors who are at the highest risk of experiencing violence. Our research offers a theoretical framework for psychological investigations into ways to improve the productivity of doctors who have experienced violence and consequent loss of motivation.

The model we created can help conduct studies that reveal society's perspective on violence against physicians and raise social awareness. It can provide an important psychological infrastructure, especially for policymakers and the prevention of violence in health care, as well as for future studies. Therefore, this study aims to introduce the schadenfreude model to measure society's perception of violence against physicians.

Our study also provides new insights into the concept of schadenfreude by contributing to the existing body of literature in a methodological aspect. In this respect, it specifically utilized structural equation modeling (SEM) to explore how well or to what extent the factors under investigation in the current research measured or affected the concept of schadenfreude after having tested the explanatory and confirmatory factor analyses. SEM refers to a series of statistical analysis techniques used to identify whether there are causal effects assumed in quantitative studies based on cross-sectional, longitudinal, experimental or other types of data, and if there is a causal effect, to estimate the magnitude and direction of this effect [50]. Bagozzi and Yi [51] pointed out that by making a clear distinction between observed and latent variables and directly taking into account measurement errors and score reliability in the model, SEM adds a more realistic dimension to the analysis, especially in the field of behavioral sciences. In our study, SEM was used to determine the level of schadenfreude regarding the violence experienced by doctors in Turkish society and to determine the causal relationships behind this emotion.

## 2 Methods

### 2.1 Research model and hypotheses

**Fig. 1:**
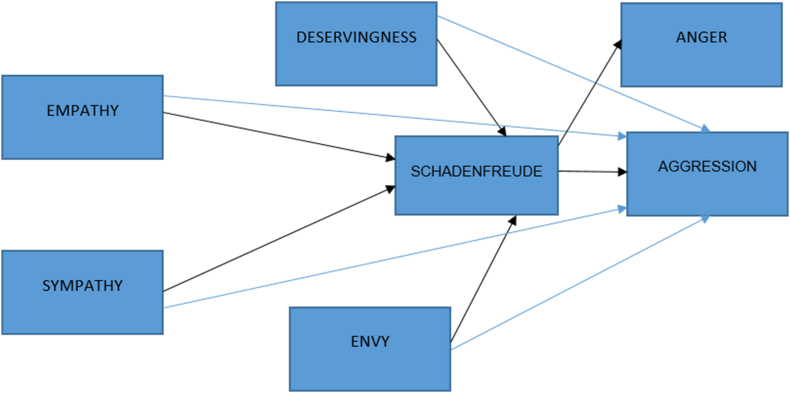
Research model.

There is limited research on schadenfreude specifically related to doctors, and studies have often used scenarios to measure schadenfreude. Our study did not require the use of scenarios, as the violence experienced in healthcare in Turkiye is widely known through the media and social media. The use of real events rather than scenarios may allow for a higher level of reliability. Furthermore, when developing the model, variables (empathy, sympathy, deservingness, envy, anger, and aggression) were added considering the previous studies. In particular, the path diagram established for other groups was used in constructing the research model [26]. The model tested in this study is presented in Fig. 1.

The following hypotheses were developed based on the literature:

H1a Empathy affects schadenfreude negatively.

H1b Sympathy affects schadenfreude negatively.

H1c Deservingness affects schadenfreude positively.

H1d Envy affects schadenfreude positively.

H2a Empathy affects aggression negatively.

H2b Sympathy affects aggression negatively.

H2c Deservingness affects aggression positively.

H2d Envy affects aggression positively.

H3a Schadenfreude affects anger positively.

H3b Schadenfreude affects aggression positively.

### 2.2 Population and sample

The population of this study consisted of people who live in the city of Erzurum, Turkiye, and do not have relatives who are primary healthcare workers and whose age was above 18. The reason why Erzurum was chosen is because it is the health center of the East Anatolian region. People from 13 provinces in the region come to the hospitals in Erzurum to receive advanced healthcare services. To represent the population of 500,000 people, it is sufficient to conduct the survey with 384 respondents with a 95 % confidence interval [52]. Moreover, it is stated that the number of people to be reached should be at least 300 and that a sample size of 50 is very poor, 100 is poor, 200 is medium, 300 is good, 500 is very good, and a sample size of 1000 or more is considered perfect [53]. According to Mundfrom et al. considering the ratio of the number of participants to the number of variables is a better way to determine the minimum sample size [54]. There is no agreed ratio in the number of samples determined by the ratio of the number of participants to the number of variables. Cattel [55] recommends three to six times the number of variables; Gorsuch [56] recommends five times the number of variables, provided it is not less than a sample size of 100; Velicer et al. [57] and Yong & Pearce [58] recommend at least five times the number of variables; and Arrindell et al. [59] recommend ten and twenty times the number of variables. This study took into account the recommendation of at least 10 times more. Ethics Committee approval was obtained from Ataturk University prior to the study (2023-17-229). A simple random sampling method was used to recruit participants, and 425 surveys were administered to patients or patient relatives whose age was above 18 and who received healthcare services at a hospital and did not have relatives who were primary care providers. Participants were informed about the purpose, scope, and confidentiality of the study prior to data collection. Research standards and ethical principles were followed, and voluntary participation was ensured by obtaining written consent forms from all participants. After excluding the incomplete surveys, a total of 402 surveys were included in the analysis.

### 2.3 Data collection tool

This study employed the survey method, one of the quantitative research methods, to collect data. After reviewing the literature, we developed a 40-item, 5-point Likert scale, with responses ranging from ‘strongly disagree’ to ‘strongly agree’ [60]. The survey consists of seven sub-scales including schadenfreude, deservingness, empathy, sympathy, anger, aggression, and envy. In order to determine the level of Schadenfreude, the malicious joy of someone else's misfortune, we used the Turkish version [61] of a four-item scale developed by Van Dijk et al. [62]. For measuring the level of deservingness, which refers to being exposed to negative behavior as a result of a situation that one deserves, we used the Turkish version [63] of a six-item scale developed by Feather [20]. For the envy dimension, which refers to the emotion that arises when a person does not possess and desires superior qualities, achievements, or possessions of another person, or wishes that the other person lacks them, we used the Turkish version [64] of a six-item scale developed by Van Dijk et al. [62]. For the dimensions of sympathy, which refers to an individual's feeling of sadness and concern for another person as a result of a bad event faced by that person, and empathy, which refers to an individual's putting himself in the other person's shoes and feeling the feelings and thoughts of the other person, we used the Turkish version [34] of a 12-item scale developed by Vossen et al. [65]. Lastly, for measuring anger, which refers to an increase in physiological arousal as a result of a threat to one's physical or psychological well-being, and aggression, which is expressed as a form of behavior aimed at harming another living being, we used a 12-item scale developed by Maxwell & Moores [66]. As a result, a 40-item pool was created.

As for content validity, the statements in the item pool were reviewed for suitability by researchers specializing in emergency medicine and psychology on the research team, sociology and psychology on comprehensibility and ease of response, linguistics and educational sciences on language and expression, and statistics on survey design and suitability for analysis, before the survey was finalized. Then, a pilot study was conducted, and the readability, comprehensibility, validity, and reliability of the scale form were checked. After the survey was administered to the participants, Cronbach Alpha coefficients were analyzed to check the reliability of the scale. As a result of the reliability analysis, the Cronbach Alpha values of schadenfreude, deservingness, envy, sympathy and empathy, anger, and aggression scales were found to be 0.961, 0.965, 0.801, 0.953, and 0.897, respectively. A Cronbach's alpha value above 0.8 indicates that the scales used are reliable [67].

## 3 Results

### 3.1 Participant demographics

The demographic distribution of participants is shown in Table 1. As shown in Table 1 below, the participants are generally between the ages of 25–34 (31.1 %), bachelor's degree holders (51.5 %), and female (63.7 %) with an income of 11,402 Turkish Liras (TL) or less (30.1 %).

**Table 1:** Participant demographics.

Variable	Category	Frequency	Percentage
Sex	Male	146	36.3
	Female	256	63.7
Age	18–24	91	22.6
	25–34	125	31.1
	35–44	94	23.4
	45–54	58	14.4
	55+	34	8.5
Marital Status	Married	210	52.2
	Single	192	47.8
Education Level	Primary Education	8	2.0
	High School	71	17.7
	Associate degree	30	7.5
	Undergraduate	207	51.5
	Graduate	86	21.4
Income Level (Monthly)	11,402 TL and below	121	30.1
	11,403 TL-20.000 TL	70	17.4
	20,001 TL-30,000 TL	119	29.6
	30,001-TL-40,000 TL	57	14.2
	40,001 TL-50,000 TL	16	4.0
	50,001 TL and above	19	4.7

Table 2 presents the correlation analysis of the variables. The correlation coefficients between r = 0.10 to 0.29 or r = −0.10 to −0.29 indicate a weak relationship; the correlation coefficients between r = 0.30 to 0.49 or r = −0.30 to −0.4.9 indicate a moderate relationship; and the correlation coefficients between r = 0.50 to 1.0 or r = −0.50 to −1.0 indicate a strong relationship among the variables [68]. Accordingly, correlation coefficients with a minus or negative sign indicate the direction of the relationship, not the strength of the relationship. According to Table 2, there is a positive correlation between deservingness and schadenfreude and a negative relationship between deservingness and envy. Moreover, the deservingness variable has a moderately negative relationship with sympathy and empathy and a strong positive relationship with both anger and aggression. Further, while there is no relationship between schadenfreude and envy, it was revealed that the schadenfreude variable has a negative relationship with sympathy and empathy and a weak positive relationship with anger and aggression. The envy variable has a weak relationship with sympathy, empathy, and anger, while it has no significant relationship with aggression. Additionally, the sympathy variable was shown to have a strong negative relationship with aggression, whereas it has a moderate negative relationship with anger. Lastly, empathy has a moderate negative relationship with anger and aggression, and the anger variable has a moderate positive relationship with aggression. The results of the correlation analysis indicated that the structural equation model could be used in the next steps of the analysis.

**Table 2:** Correlation analysis.

	Variable	Arithmetic Mean	Std. Dev.	1	2	3	4	5	6	7
1	Deservingness	1.8528	1.05611	1						
2	Schadenfreude***	1.6126	1.04030	0.171**	1					
3	Envy	2.5567	0.96182	−0.160**	0.000	1				
4	Sympathy	4.2269	0.89770	−0.473**	−0.414**	0.152**	1			
5	Empathy	3.8881	0.86443	−0.452**	−0.275**	0.293**	0.740**	1		
6	Anger	2.6950	1.05775	0.501**	0.174**	−0.104*	−0.349**	−0.332**	1	
7	Aggression	1.4075	0.67643	0.511**	0.260**	0.005	−0.583**	−0.492**	0.359**	1

*p < 0.05 **p < 0.01 *** Schadenfreude items are reverse-coded.

### 3.2 Structural equation model

The structural equation model (SEM) is considered a general model of various statistical and econometric methods, such as variance, covariance, factor, path, multiple regression analysis, simultaneous equation models, etc. SEM includes a range of multivariate statistical approaches to empirical data, both traditional and recently developed techniques. In social science research, SEM is used as an approach that combines factor analysis and simultaneous equation models. Factor analysis models test hypotheses about how well sets of observed variables in an existing data set measure the latent constructs (i.e., factors) [69]. The direct effect of schadenfreude on both anger and aggression was examined in the first model of the research (Table 3). The t-values and standardized loadings are shown in Table 3. Accordingly, schadenfreude had a direct positive effect on both anger and aggression.

**Table 3:** Structural equation model summary results (Model 1).

Concepts	Items	Standardized	T-Values
schadenfreude (sch)	sch1	0.90	36.22
	sch2	0.87	36.22
	sch3	0.92	30.51
	sch4	0.97	33.99
anger (angr)	angr1	0.87	20.29
	angr2	0.80	20.29
	angr3	0.93	27.00
	angr4	0.75	18.31
	angr5	0.77	19.29
aggression (aggr)	aggr1	0.78	25.01
	aggr2	0.80	25.01
	aggr3	0.76	16.10
	aggr4	0.90	19.75
	aggr5	0.85	19.52
	aggr6	0.75	18.09

When all variables were included in the model, the significance of the effects was examined using t-values. In this case, although deservingness did not directly affect schadenfreude, it did positively affect and increase aggression. Envy had no direct or significant effect on either schadenfreude or aggression. Sympathy and empathy significantly and negatively decreased both schadenfreude and aggression. In Model 2, although schadenfreude had a significant effect on anger, its effect on aggression became insignificant. In this case, it can be said that some of the other variables have a full mediating effect.

The standardized loadings and t-values for Model 2 are shown in Table 4.

**Table 4:** Structural equation model summary results (Model 2).

Concepts	Items	Standardized	T-Values
schadenfreude (sch)	sch1	0.90	36.24
	sch2	0.87	36.24
	sch3	0.92	30.54
	sch4	0.97	33.94
anger (angr)	angr1	0.87	20.29
	angr2	0.80	20.29
	angr3	0.93	27.00
	angr4	0.75	18.31
	angr5	0.77	19.28
aggression (aggr)	aggr1	0.79	26.10
	aggr2	0.80	26.10
	aggr3	0.76	16.48
	aggr4	0.89	20.16
	aggr5	0.84	18.71
	aggr6	0.77	16.66
deservingness (dsr)	dsr1	0.94	25.00
	dsr2	0.90	23.20
	dsr3	0.89	22.97
	dsr4	0.89	22.84
	dsr5	0.96	25.96
	dsr6	0.86	21.64
envy (envy)	envy1	0.67	14.28
	envy2	0.47	9.36
	envy3	0.81	18.01
	envy4	0.87	20.13
	envy5	0.43	8.49
sympathy (symp)	symp1	0.85	21.21
	symp2	0.93	24.52
	symp3	0.95	25.42
	symp4	0.94	24.93
	symp5	0.91	23.81
empathy (emph)	emph1	0.80	18.41
	emph1	0.62	12.89
	emph1	0.78	17.44
	emph1	0.77	17.31
	emph1	0.67	14.30

The goodness-of-fit indices for models 1 and 2 are shown in Table 5 [70,71]. In both models, the AGFI values were within the acceptable range of fit, while other fit indices showed a good fit. Therefore, both models are acceptable.

**Table 5:** Goodness of fit indices for structural equation models.

Indices	Reference Value		Model 1	Model 2
	Good fit	Acceptable fit		
X^2^/df (ki square/Degrees of Freedom)	0 < χ2/df ≤ 3	3 < χ2/df ≤ 5	2.69	2.42
RMSEA (Root Mean Square Error of Approximation)	0 ≤ RMSEA ≤0.05	0.05 ≤ RMSEA ≤0.01	0.065	0.060
AGFI (Adjusted Goodness of Fit Index)	0.90<AGFI ≤1	0.80 <AGFI ≤0.90	0.80	0.81
CFI (Comparative Fit Index)	0.95 < CFI ≤1	0.90 <CFI ≤0.94	0.97	0.98
NFI (Normed Fit Index)	0.95 < NFI ≤1	0.90 <NFI≤ 0.94	0.96	0.96
NNFI (Non-Normed Fit Index)	0.95<NNFI ≤1	0.90 <NNFI ≤0.94	0.97	0.98

While t-values above 1.96 indicate that the relationship between the variables to be measured within the scope of the model is significant at the 0.05 level, t-values above 2.576 indicate that the relationship between the variables is significant at the 0.01 level [72]. The results of hypotheses with standardized loadings and T-values for the model are shown in Table 6.

**Table 6:** Hypotheses results.

NO	HYPOTHESIS	STANDARDIZED LOADINGS	T -VALUES	RESULT
H1a:	Empathy negatively affects schadenfreude.	−0.17	−3.28	Supported
H1b:	Sympathy negatively affects schadenfreude.	−0.54	−4.78	Supported
H1c:	Deservingness positively affects schadenfreude.	−0.01	−0.10	Rejected
H1d:	Envy positively affects schadenfreude.	0.10	0.53	Rejected
H2a:	Empathy negatively affects aggression.	−0.24	−2.12	Supported
H2b:	Sympathy negatively affects aggression.	−0.33	−3.18	Supported
H2c:	Deservingness positively affects aggression.	0.22	4.53	Supported
H2d:	Envy positively affects aggression.	0.03	0.82	Rejected
H3a:	Schadenfreude positively affects anger.	0.18	3.50	Supported (models 1 and 2)
H3b:	Schadenfreude positively affects aggression.	0.29	5.59	Supported (model 1)

## 4 Discussion

Based on the analysis of survey responses, this study has documented the multi-faceted and multi-dimensional nature of the schadenfreude model, the variables of which were found to affect one another and the level of schadenfreude.

The results of our study show that as the empathy tendency, which expresses that patients or their relatives put themselves in the shoes of doctors and feel their feelings and thoughts, increases, the level of schadenfreude decreases. Further, as the empathy level of patients or their relatives increases, aggression, which is expressed as a form of behavior aimed at harming doctors, decreases. A study by Vanman [73] focused on the role of empathy in healing ongoing conflicts or hostilities. There are basically two ways to increase empathy. The first of these is to enable individuals to think about themselves by putting themselves in the shoes of group members exposed to violence. Studies show that people who take the perspective of groups experiencing violence increase their empathy and decrease their propensity to use violence [74]. The second way to increase empathy is to express in-group anger toward the external group. In this case, expressing anger can reveal intergroup empathy and reduce intergroup conflict [75]. In intergroup conflicts, a sense of empathy for the external group is an important prerequisite for reconciliation [76]. Individuals' positive thoughts about doctors can be increased by making efforts such as public service announcements, billboards, and pamphlets to increase empathy for doctors in society. In addition, in Hofstede's cultural distinction, the feminine aspect of Turkish society was found to be strong, which explains that empathic tendencies are high in the cultural characteristics of Turkish society [77]. Therefore, the practices that emphasize this empathic tendency should be utilized to prevent violence against doctors.

Analysis of data from 402 patients and/or patient relatives revealed that as the level of sympathy, which expresses patients' or patients' relatives' concern for doctors as a result of a bad event encountered by doctors, increases, the level of schadenfreude, which is used to express the malicious joy felt in the face of another person's misfortune, decreases. Moreover, as the sympathy level of patients or their relatives increases, aggression towards doctors decreases. In their study of another internal/external group other than doctors, Feather, Wenzel, & McKee developed a structural model associating schadenfreude with self-esteem, inferiority complex, malicious and good-natured jealousy, anger, perceived deservingness, and sympathy [26]. The study showed the mediating effect of deserving failure and sympathy. It was also found that malicious envy has a positive relationship with the inferiority complex and the deservingness of failure dimension of schadenfreude, and a negative relationship with sympathy.

Considering the deservingness variable, it was revealed that the increase in the level of deservingness, which is used to express being subjected to bad behavior as a result of a situation that doctors deserve, does not affect the level of schadenfreude. In contrast, thinking that doctors deserve violence increases the level of aggression of patients or their relatives. According to Colin, people do not regularly and easily take pleasure in other people's sadness. For people to experience schadenfreude, the other person does not necessarily deserve it. Therefore, the schadenfreude experienced by patients or their relatives in relation to violence by doctors may not necessarily be related to the fact that they deserved their misfortune. Schadenfreude is a feeling closer to pleasure than sadism [78]. This explains why patients or their relatives are not happy about doctors' misfortune, thinking that doctors deserve violence. However, thinking that doctors deserved violence increased aggression. When society believes that doctors deserve violence, violence against them increases and becomes normalized. Van Dijk et al. [27] examined both the concepts of deservingness and schadenfreude in their study focusing on university students. The results concluded that misfortune increases schadenfreude and that perceived deservingness has a mediating effect.

As for the envy variable, the findings of the current study show that an increase in the level of envy, which is the emotion that arises when patients and their relatives do not have the superior qualifications, achievements, or possessions of doctors and desire them or wish that they lack them, does not affect the level of schadenfreude. The increase in the level of envy towards doctors did not affect the level of schadenfreude. Similar to what other researchers in the field argue, Richard et al. [79] emphasize that it is very difficult to detect envy, as the concept of envy includes both a shameful feeling of inferiority and a feeling of hostility, and very few people want to accept this situation. In parallel with the findings of the present study, Feather & Sherman [80] found that schadenfreude was not affected by envy in their study with students. On the other hand, Sundie et al.'s [81] analysis of social media with manipulation photos and comments of premium car malfunctions indicated that envy manipulation creates jealousy and that this envy is related to schadenfreude. In this case, cultural differences related to envy may be effective. According to Hofstede's study, Turkish society has a high power distance [77]. This means that the one with a strong status is generally right. According to recent research, the profession of doctor is the profession with the highest status in Turkish society [82]. In this case, patients or relatives may have accepted the high status and did not feel schadenfreude towards doctors because of envy. The concept of envy is difficult to measure in methodological aspects; therefore, the influence of the culture prevalent in society should be taken into consideration. Results may differ in different contexts, including societies with low power distance.

Considering the anger and aggression variables, the results of the study revealed that as the level of schadenfreude increases, anger, which refers to an increase in physiological arousal as a result of a threat to the physical or psychological well-being of doctors, increases. In addition, as the level of schadenfreude increases, aggression, which is expressed as a form of behavior aimed at harming doctors, increases. It is a significant finding that schadenfreude increases both anger and aggression. Schumpe and Lafrenière [83] found that when the severity of the injury level was manipulated, both schadenfreude and sadism increased as the severity of the injury increased. A study [74] conducted with university students examined whether schadenfreude and anger had any relationship and found that anger affected schadenfreude. All these results show that criminal sanctions are not the only way to prevent violence. In order to reduce the incidents of violence against doctors, it is necessary to reduce the feeling of schadenfreude in society. Therefore, setting strategic goals by health authorities may help to increase awareness and sensitization about the negative consequences of malicious intent towards doctors, and the concept of schadenfreude should be explored from different perspectives. The results showed that violence against doctors should be resisted collectively and public awareness should be raised.

## 5 Conclusion

Grounded in a societal issue faced in Turkiye, this study touched upon violence in healthcare and examined the concept of schadenfreude through the exploration of society's perspectives, namely, the perceptions of patients and their relatives about violence towards healthcare professionals, particularly doctors. Although the concept of schadenfreude has been studied in different fields, it has not been studied in relation to violence in healthcare. By identifying the underlying causes of schadenfreude, we developed a schadenfreude model that integrated the concepts of empathy, sympathy, anger, aggression, and deservingness, which have been revealed to affect schadenfreude. Making a methodological contribution to the existing body of literature, this study utilized SEM to reveal how this set of variables measured and affected the concept of schadenfreude. For the purposes of the study, a survey was developed within the scope of this research and administered to the patients and their relatives. Based on the findings obtained from data analysis, it has been demonstrated that patients and their relatives have varying levels of schadenfreude and other psychological variables. More specifically, the lower the levels of empathy and sympathy towards doctors were, the higher the levels of both schadenfreude and aggression were. Envy had no significant effect on either schadenfreude or aggression, while deservingness directly affected aggression. The perceptions of participants regarding doctors that they deserve violence increased their aggression levels. Schadenfreude had a positive and significant effect on anger and aggression.

To conclude, the present research provided prominent evidence for the multifaceted and multidimensional nature of schadenfreude and other psychological factors such as empathy, sympathy, aggression and deservingness and their impacts as critical and influential elements on the perceptions of patients and their relatives towards the violence experienced by doctors. For this reason, it seems vital to cultivate positive attitudes and perceptions among societies, particularly patients and their relatives and it is essential to acknowledge the importance of protecting doctors by establishing the idea of not tolerating violence against doctors.

### 5.1 Limitations and future research

The current research has several limitations. Firstly, this study was conducted in Erzurum, a city in the eastern part of Turkiye, which makes it difficult to generalize the findings to other contexts. Therefore, we recommend that the model developed within the scope of this research be tested in different contexts, both across the country and in other countries where violence against doctors is at a high level. Secondly, the concepts such as envy used in the model of this study are quite difficult to measure through the use of a survey method, as they include both a shameful sense of inferiority and a sense of hostility. Therefore, experimental or neuropsychological studies are recommended for future research. Next, this research is a cross-sectional study conducted over a period. Longitudinal studies could be carried out as follow-up studies after public service announcements, the creation of volunteer platforms, awareness training, and communication training to investigate the effectiveness of these interventions. Last but not least, a model for violence in healthcare related to Schadenfreude has been developed for the first time. Other researchers could conduct studies to test the model by adding other variables such as institutionalization level of hospitals, service standards, individual and cultural differences, and similar psychological variables that are not included in this model but may affect Schadenfreude.

### 5.2 Practical and societal implications

The results of this study have several implications for practice in line with the principle of “zero tolerance to violence” of the World Health Organization. From an organizational standpoint, institutional policies can be developed with high service standards to increase the level of empathy and sympathy among patients and doctors. At the same time, training on perception management, effective communication, and methods to increase the level of sympathy can be offered for doctors to help improve and manage their relationships with their patients. From a societal standpoint, similar training sessions and/or events can be organized, and volunteer platforms can be created to raise awareness in society and convey the challenges and stress that doctors experience on a daily basis in the work environment. Moreover, health authorities can create social environments where these volunteers can experience the hard working conditions of hospitals and share this experience with other community members on online platforms.

## Data availability statement

The data from this study will be available upon request.

## Ethics and consent

Ethics Committee approval was obtained from Ataturk University Social and Human Sciences Ethics Board prior to the study with the ethics approval decision number 229 and the session number of 17 on September 14, 2023 (2023-17-229). Participants were informed about the purpose, scope, and confidentiality of the study prior to data collection. Research standards and ethical principles were followed, and voluntary participation was ensured by obtaining written consent from all participants. Specifically, they were explicitly asked if they participated in the study voluntarily at the beginning of the survey form.

## CRediT authorship contribution statement

**Fatih Yıldırım:** Writing – original draft, Visualization, Supervision, Project administration, Investigation, Formal analysis, Data curation, Conceptualization. **Zeynep Çakır:** Validation, Supervision, Project administration, Methodology, Investigation. **Sefa Özdemir:** Visualization, Validation, Resources, Methodology, Investigation, Data curation. **İnci Yılmazlı Trout:** Writing – review & editing, Writing – original draft, Visualization, Validation, Resources. **Atıf Bayramoğlu:** Validation, Supervision, Project administration, Methodology, Investigation, Conceptualization. **Oğuzhan Ekinci:** Writing – review & editing, Investigation, Formal analysis, Data curation. **Serap Atasever Belli:** Writing – review & editing, Validation, Resources, Investigation. **İkram Yusuf Yarbaşı:** Validation, Software, Methodology, Investigation, Formal analysis, Data curation. **Muhammet Mutlu:** Writing – original draft, Resources. **Rıdvan Akın:** Resources, Investigation, Conceptualization. **Burcu Yaşar:** Writing – original draft, Resources. **Seda Kayapalı Yıldırım:** Software, Investigation, Formal analysis. **Ezgi Kaşdarma:** Resources, Investigation. **Begüm Yılmazcan:** Validation, Investigation.

## Declaration of competing interest

The authors declare that they have no known competing financial interests or personal relationships that could have appeared to influence the work reported in this paper.
