# Supplementary material for: Perceptions of patients and their relatives about schadenfreude towards doctors
Source: Heliyon. 2024 Jun 20;10(13):e32983. doi: 10.1016/j.heliyon.2024.e32983 (PMC11255570; doi:10.1016/j.heliyon.2024.e32983)
Supplement: Multimedia component 1 [file mmc1.docx]

**Exploratory and Confirmatory Factor Analysis**

**Results of Exploratory Factor Analysis (EFA)**

Table 1 shows the results of the reliability analysis for the deservingness variable.

**Table 1.** Reliability analysis results for the deservingness (HE) variable.

| **Deservingness Scale Items** | | **Corrected Item-Total Correlation** | **Cronbach’s Alpha Coefficient When an Item is Removed** |
| --- | --- | --- | --- |
| HE1 | I think some doctors deserve the violence they experience. | 0.891 | 0.958 |
| HE2 | I think some doctors who were subjected to violence got what they deserved. | 0.894 | 0.958 |
| HE3 | Some doctors deserve the violent events they experience. | 0.895 | 0.958 |
| HE4 | Some doctors are responsible for the violent incidents that happen to them. | 0.896 | 0.958 |
| HE5 | Some doctors experience violence in response to their actions. | 0.914 | 0.956 |
| HE6 | Some doctors experience violent incidents as a result of their own behavior. | 0.860 | 0.962 |
| **Cronbach’s Alpha** | | | **0.965** |

According to Table 1, the Cronbach’s alpha value of the deservingness scale, consisting of six items, is 0.965, which indicates that the reliability of the deservingness scale is high. When the item-total statistics are examined, if any item is removed, the Cronbach’s alpha coefficient will not increase; on the contrary, it will decrease. In the corrected item-total correlation, it is seen that all values ​​are greater than 0.30.

**Table 2.** Reliability Analysis for the Schadenfreude (SF) Variable

| **Schadenfreude Scale Items** | | **Corrected Item-Total Correlation** | **Cronbach’s Alpha Coefficient When an Item is Removed** |
| --- | --- | --- | --- |
| SF1 | I do not rejoice in the violence that happens to doctors. | 0.910 | 0.946 |
| SF2 | When I hear about violent incidents involving doctors, I don’t say ‘it serves him/her right’. | 0.896 | 0.951 |
| SF3 | Violence that happens to doctors does not make me happy. | 0.894 | 0.951 |
| SF4 | When I hear about violent incidents involving doctors, I don’t say ‘I’m glad it happened.’ | 0.915 | 0.945 |
| **Cronbach’s Alpha** | | | **0.961** |

According to Table 2, the Cronbach’s alpha value of the Schadenfreude scale, consisting of four items, is 0.961, which indicates that the reliability of the Schadenfreude scale is high. When the item-total statistics are examined, if any item is removed, the Cronbach’s alpha coefficient will not increase; on the contrary, it will decrease. In the corrected item-total correlation, it is seen that all values ​​are greater than 0.30.

**Table 3.** Reliability Analysis for the Envy (KI) Variable

| **Envy Scale Items** | | **Corrected Item-Total Correlation** | **Cronbach’s Alpha Coefficient When an Item is Removed** |
| --- | --- | --- | --- |
| KI1 | I would have wanted to be a doctor. | 0.611 | 0.755 |
| KI2 | I often envy doctors. | 0.505 | 0.787 |
| KI3 | I would want to have the dignity of a doctor. | 0.648 | 0.742 |
| KI5 | I would like to have the status of a doctor. | 0.728 | 0.713 |
| KI6 | I feel bad when I compare myself to doctors in terms of status. | 0.441 | 0.803 |
| **Cronbach’s Alpha** | | | **0.801** |

According to Table 3, in the corrected item-total correlation of the envy scale consisting of 6 items, as the item "I get angry with doctors" (K4) had a correlation coefficient of 0.051, which is less than 0.30, the relevant item was not included in the analysis. Then, a Cronbach’s alpha value of 0.801 was obtained, which indicates that the reliability of the envy scale is sufficient. In the corrected item-total correlation, it is seen that all values ​​are greater than 0.30.

**Table 4.** Reliability Analysis for the Sympathy (S) and Empathy (E) Variables

| **Sympathy and Empathy Scale Items** | | **Corrected Item-Total Correlation** | **Cronbach’s Alpha Coefficient When an Item is Removed** |
| --- | --- | --- | --- |
| S1 | When doctors feel sad about being subjected to violence, I feel sad too. | 0.819 | 0.947 |
| S2 | I feel sorry for a doctor who was subjected to violence. | 0.842 | 0.947 |
| S3 | I worry about doctors who get hurt by violence. | 0.859 | 0.946 |
| S4 | When doctors who are subjected to violence get upset, I get upset too. | 0.844 | 0.946 |
| S5 | I worry about doctors who are subjected to violence. | 0.828 | 0.947 |
| E1 | Even if I don't witness an act of violence towards doctors, I can understand how they feel. | 0.768 | 0.949 |
| E2 | I can easily describe how doctors who experience violence feel. | 0.587 | 0.955 |
| E3 | It is understandable that the doctor who was subjected to violence would be angry. | 0.742 | 0.950 |
| E4 | It is understandable that the doctor who is subjected to violence is not actually happy. | 0.744 | 0.949 |
| E5 | I get angry when doctors are violent. | 0.853 | 0.946 |
| E6 | I get scared when doctors are subjected to violence. | 0.638 | 0.954 |
| E7 | I get angry when doctors experience acts of violence. | 0.820 | 0.947 |
| **Cronbach’s Alpha** | | | **0.953** |

According to Table 4, the Cronbach’s alpha value of the sympathy and empathy scale, consisting of 12 items, is 0.953, which indicates that the reliability of the sympathy and empathy scale is high. In the corrected item-total correlation, it is seen that all values ​​are greater than 0.30.

**Table 5.** Reliability Analysis of the Anger (K) and Aggression (SA) Variable

| **Anger and Aggression Scale Items** | | **Corrected Item-Total Correlation** | **Cronbach’s Alpha Coefficient When an Item is Removed** |
| --- | --- | --- | --- |
| K1 | I feel angry towards the attitudes and behaviors of doctors. | 0.677 | 0.886 |
| K2 | I feel resentful towards the attitudes and behaviors of doctors. | 0.580 | 0.892 |
| K3 | I get angry at doctors' attitudes and behavior. | 0.719 | 0.883 |
| K4 | I show my anger towards the attitudes and behaviors of doctors. | 0.632 | 0.889 |
| K5 | I have difficulty controlling my anger towards doctors' attitudes and behaviors. | 0.587 | 0.891 |
| K6 | Doctors' attitudes and behaviors make me angry. | 0.720 | 0.883 |
| SA1 | Violent behavior towards doctors is acceptable. | 0.618 | 0.890 |
| SA2 | Using physical force against doctors is acceptable. | 0.574 | 0.892 |
| SA3 | When I get angry at a doctor, I get sarcastic with her/him. | 0.583 | 0.891 |
| SA4 | I use force against doctors to take my frustration out. | 0.572 | 0.893 |
| SA5 | When I get angry at doctors, I verbally insult them. | 0.567 | 0.892 |
| SA6 | Violence against doctors is acceptable to a certain degree. | 0.642 | 0.888 |
| **Cronbach’s Alpha** | | | **0.897** |

Table 5 shows that the Cronbach’s alpha value of the 12-item anger and aggression scale is 0.897, which indicates that the anger and aggression scale is reliable. When the item-total statistics are examined, if any item is removed, the Cronbach’s alpha coefficient will not increase; on the contrary, it will decrease. In the corrected item-total correlation, it is seen that all values ​​are greater than 0.30.

**Table 6.** Exploratory Factor Analysis of the Deservingness (HE) Variable

| **Items** | **Factor Loadings** | **Explained Variance** | **KMO** |
| --- | --- | --- | --- |
| HE1 | 0.926 | 85.763 | 0.900 |
| HE2 | 0.930 |  |  |
| HE3 | 0.931 |  |  |
| HE4 | 0.927 |  |  |
| HE5 | 0.941 |  |  |
| HE6 | 0.901 |  |  |
| **Bartlett’s Test of Sphericity (p)** | | | **0.000** |

According to Table 6, the KMO value of six items regarding the deservingness scale is 0.900, which indicates that the variables are suitable for factor analysis. The Bartlett’s Test of Sphericity was p (Sig)=0.00 <0.05. Therefore, the data are suitable for factor analysis. The deservingness scale consists of six items and one dimension, and this dimension explains 85.763% of the variance.

**Table 7.** Factor Analysis of the Schadenfreude (SF) Variable

| **Items** | **Factor Loadings** | **Explained Variance** | **KMO** |
| --- | --- | --- | --- |
| SF1 | 0.950 | 89.559 | 0.844 |
| SF2 | 0.942 |  |  |
| SF3 | 0.941 |  |  |
| SF4 | 0.953 |  |  |
| **Bartlett’s Test of Sphericity (p)** | | | **0.000** |

Table 7 shows that the KMO value of four items related to the Schadenfreude scale is 0.844, which indicates that the variables are suitable for factor analysis. The Bartlett's Test of Sphericity was p (Sig)=0.00 <0.05. Therefore, the data are suitable for factor analysis. The Schadenfreude scale consists of 4 items and one dimension, and this dimension explains approximately 89.559% of the total variance.

**Table 8.** Factor Analysis of the Envy (KI) Variable

| **Items** | **Factor Loadings** | **Explained Variance** | **KMO** |
| --- | --- | --- | --- |
| KI1 | 0.772 | 55.865 | 0.773 |
| KI2 | 0.670 |  |  |
| KI3 | 0.801 |  |  |
| KI5 | 0.855 |  |  |
| KI6 | 0.612 |  |  |
| **Bartlett’s Test of Sphericity (p)** | | | **0.000** |

Table 8 shows that the KMO value of 5 items regarding the envy scale is 0.773, which indicates that the variables are suitable for factor analysis. The Bartlett’s Test of Sphericity was p (Sig)=0.00 <0.05. Therefore, the data are suitable for factor analysis. The envy scale consists of five items and one dimension, and this dimension explains approximately 55.865% of the variance.

**Table 9.** Factor Analysis of the Sympathy (S) and Empathy (E) Variables

| **Items** | **Factor Loadings** | **Explained Variance** | **KMO** |
| --- | --- | --- | --- |
| **Factor 1: Sympathy** | | 66.802 | 0.920 |
| S4 | 0.888 |  |  |
| S2 | 0.888 |  |  |
| S3 | 0.882 |  |  |
| S5 | 0.855 |  |  |
| S1 | 0.807 |  |  |
| **Factor 2: Empathy** | | 10.229 |  |
| E2 | 0.845 |  |  |
| E1 | 0.809 |  |  |
| E3 | 0.735 |  |  |
| E4 | 0.650 |  |  |
| E6 | 0.540 |  |  |
| **Bartlett’s Test of Sphericity (p) 0.000** | | **Total Explained Variance** | **77.031** |

In the exploratory factor analysis conducted on the sympathy and empathy scale, two items - 'I get angry when doctors experience violence.' (E5) and 'I get upset when doctors experience violence.' (E7)- were not included in the analysis as they cross-loaded with a cross-load value that is less than 0.10. Then, an EFA was completed, which showed that the KMO value of 10 items related to the sympathy and empathy scale was 0.920. This value shows that the variables are suitable for factor analysis. The Bartlett's Test of Sphericity was p (Sig)=0.00 <0.05. Therefore, the data are suitable for factor analysis. The 10-item sympathy and empathy scale consists of two dimensions, sympathy and empathy, which explain 77.031% of the total variance. The sympathy dimension explains 66.802% of the total variance, and the empathy dimension explains 10.229% of the total variance. The dimensional distribution of the items on this scale shows similar distributions to the studies conducted by Vossen et al., (2015) and Zengin et al., (2018).

**Table 10.** Factor Analysis of the Anger (K) and Aggression (SA) Variables

| **Items** | **Factor Loadings** | **Explained Variance** | **KMO** |
| --- | --- | --- | --- |
| **Factor 1: Aggression** | | 49.750 | 0.892 |
| SA4 | 0.900 |  |  |
| SA2 | 0.867 |  |  |
| SA1 | 0.850 |  |  |
| SA5. | 0.848 |  |  |
| SA6 | 0.779 |  |  |
| SA3 | 0.768 |  |  |
| **Factor 2: Anger** | | 24.044 |  |
| K3 | 0.918 |  |  |
| K1. | 0.887 |  |  |
| K2 | 0.847 |  |  |
| K6 | 0.796 |  |  |
| K4 | 0.791 |  |  |
| **Bartlett’s Test of Sphericity (p) 0.000** | | **Total Explained Variance** | **73.794** |

In the exploratory factor analysis conducted on the anger and aggression scale, the item "I have difficulty controlling my anger towards the attitudes and behaviors of doctors" (K5) was not included in the analysis as it cross-loaded with a value that was less than 0.10. The subsequent EFA showed that the KMO value of 11 items related to the anger and aggression scale was 0.892. This value shows that the variables are suitable for factor analysis. The Bartlett’s Test of Sphericity was p (Sig)=0.00 <0.05. Therefore, the data are suitable for factor analysis. The anger and aggression scale consists of 11 items and two dimensions – anger and aggression-, and these dimensions explain 73.794% of the total variance. The anger dimension explains 49.750% of the total variance, and the empathy dimension explains 24.044% of the total variance. The dimensional distribution of the items on this scale shows similar distributions to the study conducted by Maxwell and Moores (2007).

**Results of the Confirmatory Factor Analysis (CFA)**

Confirmatory factor analysis defines each observed variable only under its own latent variable and describes the relationship between factors (Çokluk et al., 2014). The reason for using this analysis at this stage is that it is necessary to test the validity of the variables before they are included in the determined structural model to obtain the most appropriate measurement model. In this context, it is aimed to verify the factor structures of the scales based on the goodness-of-fit statistics included in the structural equation model (Hair et al., 2014). Among the items with factor loadings, those with standard factor loadings of 0.50 and above are considered important items for the scale, and those with factor loadings below 0.30 are not included in the analysis (Güngören et al., 2014, p.74; Jöreskog and Sörbom, 1996). On the other hand, in CFA, some values, such as CFI, IFI, NFI, NNFI, etc., ​​must be within the range of fit indices (Yıldırım and Naktiyok, 2017). The reference range of these fit indices is given in the table below, along with the indices of our research. For reference values, the study of Meydan and Şeşen (2011) was used.

**Table 11.** Fit Indices of the Confirmatory Factor Analysis (CFA)

| **Indices** | **Reference Value** | | **HE** | **SF** | **KIS** | **S-E** | **K-S** |
| --- | --- | --- | --- | --- | --- | --- | --- |
|  | **Good fit** | **Acceptable Fit** |  |  |  |  |  |
| X^2^/SD | 0<χ2/sd ≤ 3 | 3<χ2/sd ≤ 5 | 1.98 | 0.00 | 4.31 | 3.8 | 4.77 |
| RMSEA | 0 ≤ RMSEA ≤ 0,05 | 0,05 ≤ RMSEA ≤ 0,01 | 0.049 | 0.00 | 0.091 | 0.084 | 0.097 |
| GFI | 0,95<GFI ≤ 1 | 0,90 <GFI ≤ 0,94 | 0.99 | - | 0.98 | 0.94 | 0.92 |
| AGFI | 0,90<AGFI ≤ 1 | 0,80 <AGFI ≤ 0,90 | 0.97 | - | 0.94 | 0.90 | 0.87 |
| CFI | 0,95<CFI ≤ 1 | 0,90 <CFI ≤ 0,94 | 1 | - | 0.98 | 0.99 | 0.97 |
| NFI | 0,95<NFI ≤ 1 | 0,90 <NFI≤ 0,94 | 1 | - | 0.98 | 0.98 | 0.96 |
| NNFI | 0,95<NNFI ≤ 1 | 0,90 <NNFI ≤ 0,94 | 1 |  | 0.96 | 0.98 | 0.96 |

In Table 11, the goodness of fit indices of the confirmatory factor analysis are presented, and the goodness of fit measures of the factors are given. In the table, the deservingness variable is indicated by HE, the schadenfreude variable is indicated by SF, the envy variable is indicated by KIS, the sympathy and empathy variables are indicated by S-E, and the anger and aggression variables are indicated by K-S. The χ2/df=1.98, RMSEA=0.049, GFI= 0.99, AGFI=0.97, CFI=1, NFI=1, NNFI=1 values ​​of the deservingness dimension are all within the range of good fit measures. The χ2/sd=0.00 and RMSEA=0.00 values ​​of the Schadenfreude dimension are within the range of good fit indices. However, it was concluded that the fit of the schadenfreude dimension was excellent. χ2/df=4.31 and RMSEA=0.091 values ​​of the envy dimension are within acceptable fit measures, while GFI=.98, AGFI=.94, CFI=0.98, NFI=0.98, and NNFI=0.96 values ​​are within the range of good fit indices. The values ​​of χ2/df=3.8, RMSEA=0.084, GFI=0.94, and AGFI=0.90 of the sympathy and empathy dimension are within the acceptable fit indices, and the values of CFI=0.99, NFI=0.98, and NNFI=0.98 are within the range of good fit indices. The χ2/sd=4.77, RMSEA=0.097, GFI= 0.92, and AGFI=0.87 values ​​of the anger and aggression dimension are within the acceptable fit indices, and the CFI=0.97, NFI=0.96, and NNFI=0.96 values ​​are within the range of good fit indices. In alignment with the CFA results, the obtained values ​​are within the acceptable range compared to the reference values ​​shown in Table 13. As a result of the confirmatory factor analysis, LISREL outputs of standard factor loadings are given in the figures below.

**Figure 1.** LISREL Output of the Standardized Loadings of the Deservingness (HakEtme) Dimension Confirmatory Factor Analysis


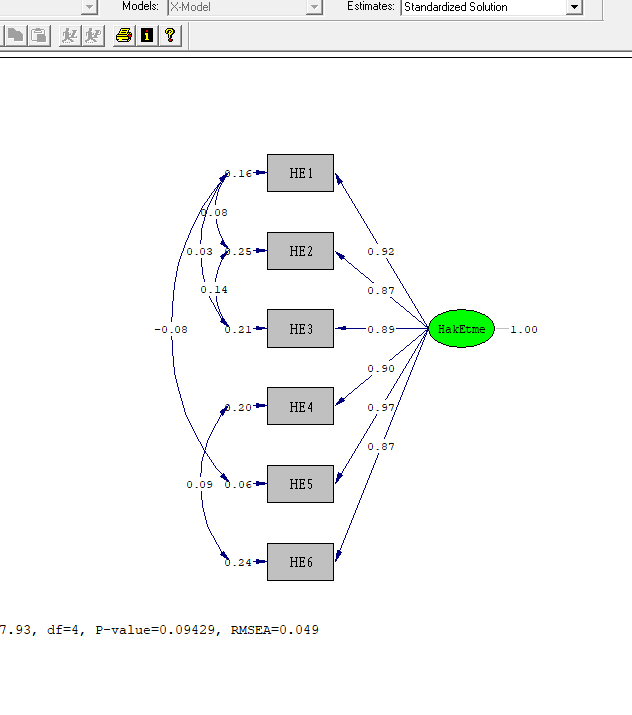


In confirmatory factor analysis deservingness variable coded as “Haketme”. As per the guidance of the LISREL program, modifications were made by connecting the items HE2 and HE3, HE1 and HE2, HE1 and HE3, H1 and H3, HE1 and HE5, and HE6 and HE4.

**Figure 2.** LISREL Output of the Standardized Loadings of the Schadenfreude Dimension Confirmatory Factor Analysis


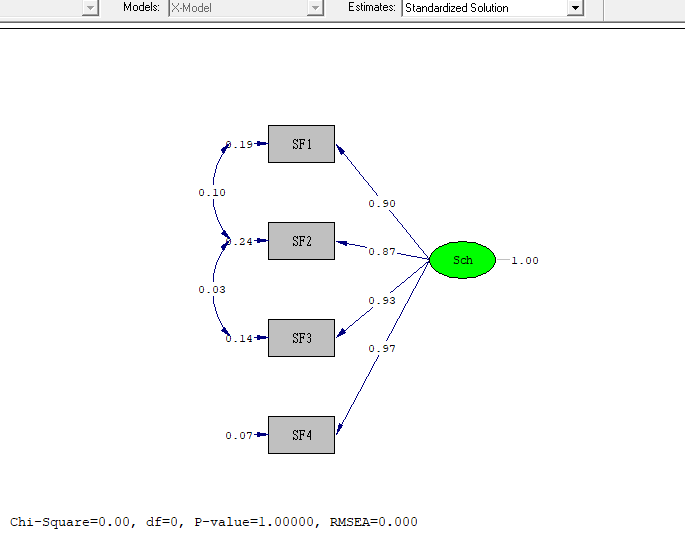


In confirmatory factor analysis schadenfreude variable coded as “Sch”. As per the guidance of the LISREL program, modifications were made by connecting the items SF1 and SF2 and SF3 and SF2.

**Figure 3.** LISREL Output of the Standardized Loadings of the Envy (Kis) Dimension Confirmatory Factor Analysis


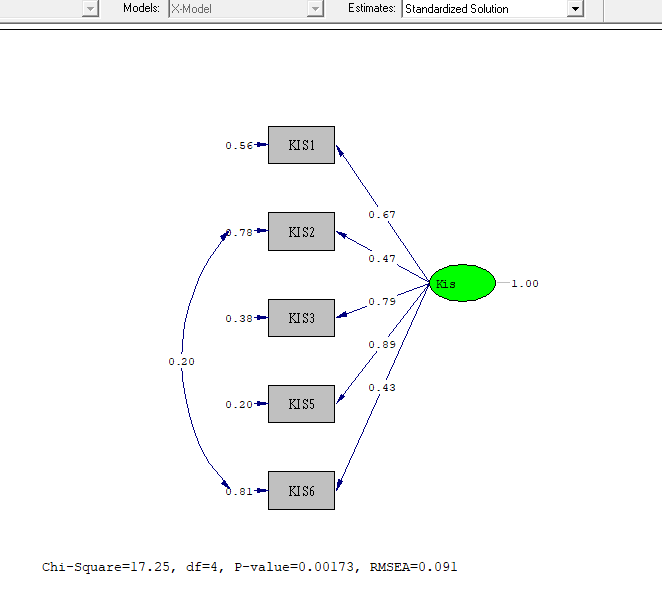


In confirmatory factor analysis envy variable coded as “Kis”. As per the guidance of the LISREL program, modifications were made by connecting the KIS2 and KIS6 items to each other.

**Figure 4.** LISREL Output of the Standardized Loadings of the Sympathy (Sem) and Empathy (Emp) Dimension Confirmatory Factor Analysis


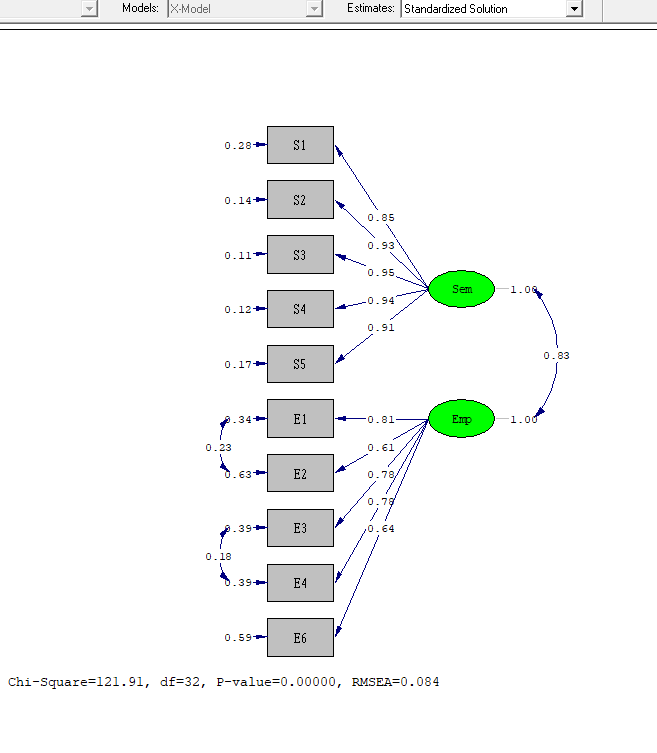


In confirmatory factor analysis sympathy variable coded as “Sem” and empathy variable coded as “Emp”. As per the guidance of the LISREL program, modifications were made by connecting the items E1 and E2 and E3 and E4 together.

**Figure 5.** LISREL Output of the Standardized Loadings of the anger (kzg) and aggression (sldr) Dimension Confirmatory Factor Analysis


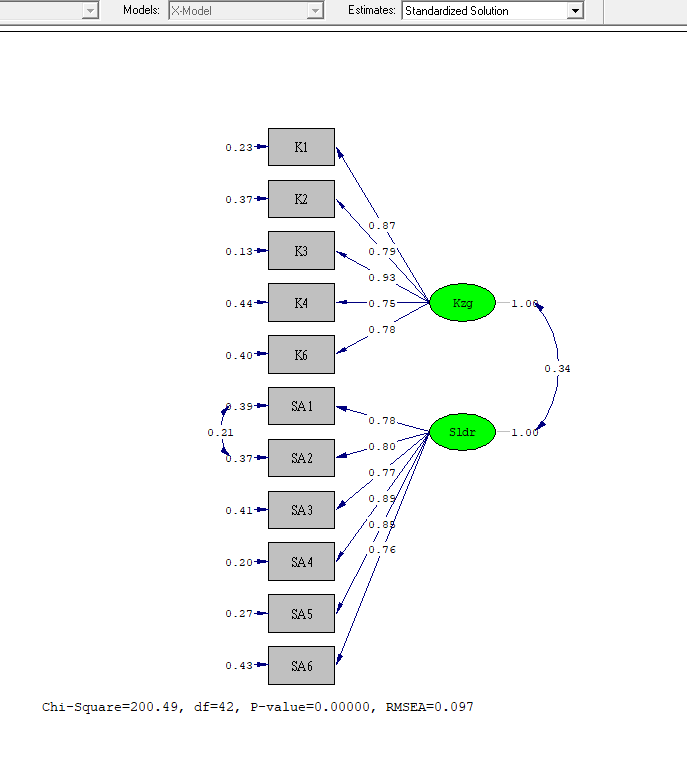


In confirmatory factor analysis, anger variable coded as “Kzg” and aggression variable coded as “Sldr”. As per the guidance of the LISREL program, modifications were made by connecting SA1 and SA2 items. According to the confirmatory factor analysis, no standardized factor loading is below 0.30 or above 1. Therefore, no items were removed at this stage. Standard factor loadings and t-values ​​for confirmatory factor analysis are summarized in Table 12.

**Table 12.** A Summary of the Results Related to the Concepts in the Model

| **Concept in the Model** | **Items** | **Standard Values** | **T Values** |
| --- | --- | --- | --- |
| **DESERVINGNESS** | HE1 | 0.92 | 23.22 |
|  | HE2 | 0.87 | 21.73 |
|  | HE3 | 0.89 | 22.58 |
|  | HE4 | 0.90 | 22.89 |
|  | HE5 | 0.97 | 26.30 |
|  | HE6 | 0.87 | 21.82 |
| **SCHADENFREUDE** | SF1 | 0.90 | 23.21 |
|  | SF2 | 0.87 | 21.85 |
|  | SF3 | 0.93 | 24.20 |
|  | SF4 | 0.97 | 26.07 |
| **ENVY** | KIS1 | 0.67 | 14.13 |
|  | KIS2 | 0.47 | 9.34 |
|  | KIS3 | 0.79 | 17.42 |
|  | KIS5 | 0.89 | 20.47 |
|  | KIS6 | 0.43 | 8.53 |
| **SYMPATHY** | S1 | 0.85 | 21.17 |
|  | S2 | 0.93 | 24.55 |
|  | S3 | 0.95 | 25.33 |
|  | S4 | 0.94 | 25.01 |
|  | S5 | 0.91 | 23.79 |
| **EMPATHY** | E1 | 0.81 | 18.59 |
|  | E2 | 0.61 | 12.55 |
|  | E3 | 0.78 | 17.54 |
|  | E4 | 0.76 | 17.46 |
|  | E6 | 0.64 | 13.62 |
| **ANGER** | K1 | 0.87 | 21.81 |
|  | K2 | 0.79 | 18.79 |
|  | K3 | 0.93 | 24.30 |
|  | K4 | 0.75 | 17.29 |
|  | K6 | 0.78 | 18.14 |
| **AGGRESSION** | SA1 | 0.78 | 18.19 |
|  | SA2 | 0.80 | 18.69 |
|  | SA3 | 0.77 | 17.77 |
|  | SA4 | 0.89 | 22.37 |
|  | SA5 | 0.88 | 20.75 |
|  | SA6 | 0.76 | 17.39 |

As presented in Tables 11 and 12, standard loadings, t-values, ​​and fit indices are within acceptable value ranges.
