# Supplementary material for: Perceptions of patients and their relatives about schadenfreude towards doctors
Source: Heliyon. 2024 Jun 20;10(13):e32983. doi: 10.1016/j.heliyon.2024.e32983 (PMC11255570; doi:10.1016/j.heliyon.2024.e32983)
Supplement: Multimedia component 2 [file mmc2.docx]

**Survey Form**

Dear Participants,

Confidentiality: Your participation in this research study is voluntary, and if you decide not to participate in this research survey, you may withdraw at any time. Your responses will be kept confidential, and we do not collect identifying information such as your name, or e-mail address. Your responses will never be used in a way that can identify you individually. There are no right or wrong answers to the questions. Therefore, it is very important not to leave the questions blank in order to obtain reliable information from the results of the research. These questionnaire items will take approximately 10 minutes.

Thank you for your participation.

For more information about the study, please feel free to contact Assoc. Prof. Dr. Fatih Yildirim ([fatih.yildirim@erzurum.edu.tr](mailto:fatih.yildirim@erzurum.edu.tr))

I voluntarily participate in this study. □

| **Deservingness Scale Items** | |
| --- | --- |
| Dsr1 | I think some doctors deserve the violence they experience. |
| Dsr2 | I think some doctors who were subjected to violence got what they deserved. |
| Dsr3 | Some doctors deserve the violent events they experience. |
| Dsr4 | Some doctors are responsible for the violent incidents that happen to them. |
| Dsr5 | Some doctors experience violence in response to their actions. |
| Dsr6 | Some doctors experience violent incidents as a result of their own behavior. |
| **Schadenfreude Scale Items** | |
| Sch1 | I do not rejoice in the violence that happens to doctors. |
| Sch2 | When I hear about violent incidents involving doctors, I don’t say ‘it serves him/her right’. |
| Sch3 | Violence that happens to doctors does not make me happy. |
| Sch4 | When I hear about violent incidents involving doctors, I don’t say ‘I’m glad it happened.’ |
| **Envy Scale Items** | |
| Envy1 | I would have wanted to be a doctor. |
| Envy2 | I often envy doctors. |
| Envy3 | I would want to have the dignity of a doctor. |
| Envy5 | I would like to have the status of a doctor. |
| Envy6 | I feel bad when I compare myself to doctors in terms of status. |
| **Sympathy Scale Items** | |
| Symp1 | When doctors feel sad about being subjected to violence, I feel sad too. |
| Symp2 | I feel sorry for a doctor who was subjected to violence. |
| Symp3 | I worry about doctors who get hurt by violence. |
| Symp4 | When doctors who are subjected to violence get upset, I get upset too. |
| Symp5 | I worry about doctors who are subjected to violence. |
| **Empathy Scale Items** | |
| Emph1 | Even if I don't witness an act of violence towards doctors, I can understand how they feel. |
| Emph2 | I can easily describe how doctors who experience violence feel. |
| Emph3 | It is understandable that the doctor who was subjected to violence would be angry. |
| Emph4 | It is understandable that the doctor who is subjected to violence is not actually happy. |
| Emph6 | I get scared when doctors are subjected to violence. |
| **Anger Scale Items** | |
| Angr1 | I feel angry towards the attitudes and behaviors of doctors. |
| Angr2 | I feel resentful towards the attitudes and behaviors of doctors. |
| Angr3 | I get angry at doctors' attitudes and behavior. |
| Angr4 | I show my anger towards the attitudes and behaviors of doctors. |
| Angr6 | Doctors' attitudes and behaviors make me angry. |
| **Aggression Scale Items** | |
| Aggr1 | Violent behavior towards doctors is acceptable. |
| Aggr2 | Using physical force against doctors is acceptable. |
| Aggr3 | When I get angry at a doctor, I get sarcastic with her/him. |
| Aggr4 | I use force against doctors to take my frustration out. |
| Aggr5 | When I get angry at doctors, I verbally insult them. |
| Aggr6 | Violence against doctors is acceptable to a certain degree. |
